# Association of Prospective Falls in Older People With Ubiquitous Step-Based Fall Risk Parameters Calculated From Ambulatory Inertial Signals: Secondary Data Analysis

**DOI:** 10.2196/49587

**Published:** 2023-11-24

**Authors:** Nahime Al Abiad, Kimberley S van Schooten, Valerie Renaudin, Kim Delbaere, Thomas Robert

**Affiliations:** 1 Laboratoire de Biomécanique et Mécanique des Chocs, Université Gustave Eiffel and Université Claude Bernard Lyon 1, Lyon, France; 2 Laboratoire de Géolocalisation, Université Gustave Eiffel, Bouguenais, France; 3 Falls, Balance and Injury Research Centre, Neuroscience Research Australia, Randwick, Australia; 4 School of Population Health, University of New South Wales, Kensington, Australia

**Keywords:** fall risk biomarkers, prospective falls, sensor placement, inertial measurement units, fall prediction, older adults, older adult, geriatric, geriatrics, elderly, fall, sensor, sensors, inertial measurement, model, predict, prediction, predictive

## Abstract

**Background:**

In recent years, researchers have been advocating for the integration of ambulatory gait monitoring as a complementary approach to traditional fall risk assessments. However, current research relies on dedicated inertial sensors that are fixed on a specific body part. This limitation impacts the acceptance and adoption of such devices.

**Objective:**

Our study objective is twofold: (1) to propose a set of step-based fall risk parameters that can be obtained independently of the sensor placement by using a ubiquitous step detection method and (2) to evaluate their association with prospective falls.

**Methods:**

A reanalysis was conducted on the 1-week ambulatory inertial data from the *StandingTall* study, which was originally described by Delbaere et al. The data were from 301 community-dwelling older people and contained fall occurrences over a 12-month follow-up period. Using the ubiquitous and robust step detection method *Smartstep,* which is agnostic to sensor placement, a range of step-based fall risk parameters can be calculated based on walking bouts of 200 steps. These parameters are known to describe different dimensions of gait (ie, variability, complexity, intensity, and quantity). First, the correlation between parameters was studied. Then, the number of parameters was reduced through stepwise backward elimination. Finally, the association of parameters with prospective falls was assessed through a negative binomial regression model using the area under the curve metric.

**Results:**

The built model had an area under the curve of 0.69, which is comparable to models exclusively built on fixed sensor placement. A higher fall risk was noted with higher gait variability (coefficient of variance of stride time), intensity (cadence), and quantity (number of steps) and lower gait complexity (sample entropy and fractal exponent).

**Conclusions:**

These findings highlight the potential of our method for comprehensive and accurate fall risk assessments, independent of sensor placement. This approach has promising implications for ambulatory gait monitoring and fall risk monitoring using consumer-grade devices.

## Introduction

Falls in older people are a major cause of mobility loss, morbidity, and mortality. With over one-third of people aged 65 years and older falling at least once a year [1], identifying individuals at risk and providing them with appropriate interventions is crucial [2]. However, traditional approaches typically rely on a single clinical evaluation session (eg, St Thomas's Risk Assessment Tool in Falling Elderly Inpatients [3] or the Balance Evaluation Systems Test and its variants [4]) and have demonstrated inconsistent and limited predictive power. Therefore, recent studies have moved toward continuous monitoring approaches. For example, positive results were obtained using electronic health records from long-term care facility residents [5]. However, this approach is restricted to a specific population in a specific setting and, therefore, might not apply to the general population.

Fall risk biomarkers based on ambulatory gait monitoring are increasingly used to complement the initial clinical evaluations [6-10]. Such daily-life gait monitoring offers improved performance of fall prediction by incorporating daily-life gait, which enables the assessment of participants’ actual gait performance in real-life situations rather than solely in laboratory settings [9,11,12]. For example, van Schooten et al [8] showed that adding 7-day ambulatory measures to clinical measures increases the ability of the model to discriminate people who sustained a fall during the 12-month follow-up period from those who did not: the area under the curve (AUC) increased from 0.68 to 0.82.

Numerous models have been proposed for predicting falls based on sensor-based data. However, the vast majority of these models lack essential properties required for generalizability [13]. Only a few study remained [8,10,14,15] when focusing exclusively on studies that meet three criteria: (1) reliance on ambulatory or real-life inertial data collected from a single unit, (2) the use of prospective falls collected during a follow-up period as a criteria to identify fallers, and (3) the inclusion of a sufficient population of community-dwelling older people (>100). These studies draw from a similar data set to the one used in this study, but they differ slightly in terms of the types of fall predictors (different gait domains), modeling approaches (binomial regressions with extreme or median value of predictors [8,10], survivor analysis [14], and the use of deep learning techniques [15]), and subsets of the database used. The resulting AUC ranged from 0.71, when using only accelerometric data [8], to as high as 0.74, when selecting gait data samples [15]; the AUC was even higher when incorporating clinical variables (0.81-0.82) [8,10]. These results showed that fall risk can be predicted with a reasonable accuracy when using ambulatory gait data obtained with a single inertial measurement unit placed on the lower torso.

Consumer-grade devices, such as smartphones and smartwatches, almost systematically embed inertial measurement unit sensors whose quality is largely sufficient for gait monitoring applications (eg, the study by Manor et al [16]). More importantly, they are well accepted and already widely available. The integration of gait monitoring into consumer-grade devices would enable the monitoring of fall risk on a large scale, which could lead to a substantial improvement in fall risk identification and subsequent prevention. However, up until now, ambulatory gait monitoring of fall risk parameters has been limited to a dedicated inertial sensor and a fixed body placement, usually on the trunk or feet. Switching to consumer-grade device monitoring presents several challenges. Some issues relate to the devices themselves, such as memory and battery use. However, one of the most critical challenges is to develop a gait-monitoring approach that can withstand the flexibility of consumer-grade device placement (eg, wrist or pocket) and carrying modes for handheld devices (eg, swinging, hand in pocket, or texting). Indeed, imposing a fixed body position or type of motion strongly limits the utility of the approach [17]. A ubiquitous solution should be (1) predictive of prospective falls, (2) independent of sensor placement, and (3) modest in terms of data processing (ie, computational cost, size of data set, and complexity of method).

One of the key issues to making the solution ubiquitous is the type of parameters from which fall risk is estimated. Most fall prediction models rely on parameters estimated from the inertial sensor time series, such as the Lyapunov exponents [8,10]. These *signal-based* parameters are indeed relatively straightforward to calculate. However, a major drawback is that they are affected by sensor placement both in terms of value and the strength of their association with the risk of falls [18,19].

In this context, fall risk estimated using parameters calculated based on step instants offers potential, with promising results in laboratory settings and pathological gait [20-23]. Importantly, there exists a robust step detection method that allows the estimation of these step instants independently of sensor placement. An example is the recent *Smartstep* algorithm [24], relying on 2 machine learning models (1 for the gyroscope and 1 for the accelerometer data) plus a decision process that determines which machine learning model is to be used as the main step detector. Its robustness against sensor body placements (waist, pants or jacket pockets, or handheld), different walking conditions, and real-life challenges (eg, blind people walking outside with or without aids, older adults in a hospital hallway open to the public, etc) has been demonstrated [24,25]. Using Smartstep, it is thus possible to estimate some *step-based* parameters independently of the sensor placement. They can be gait variability indicators such as the SD and coefficient of variance of stride time, gait complexity indicators such as fractal exponent and sample entropy of stride time, gait intensity indicators such as cadence, and gait quantity indicators such as the total number of steps. Demonstrating that fall risk can be assessed using such *step-based* parameters calculated from ambulatory data could be a decisive step toward a ubiquitous fall risk prediction solution that is insensitive to device location.

Therefore, this study aims to evaluate the association of *step-based* parameters, calculated using the ubiquitous step detection method Smartstep, and prospective falls [24]. The study is a secondary analysis using a data set collected by Delbaere et al [26]. It involves ambulatory gait inertial signals from the lower back.

## Methods

### Overview

**Figure 1.**
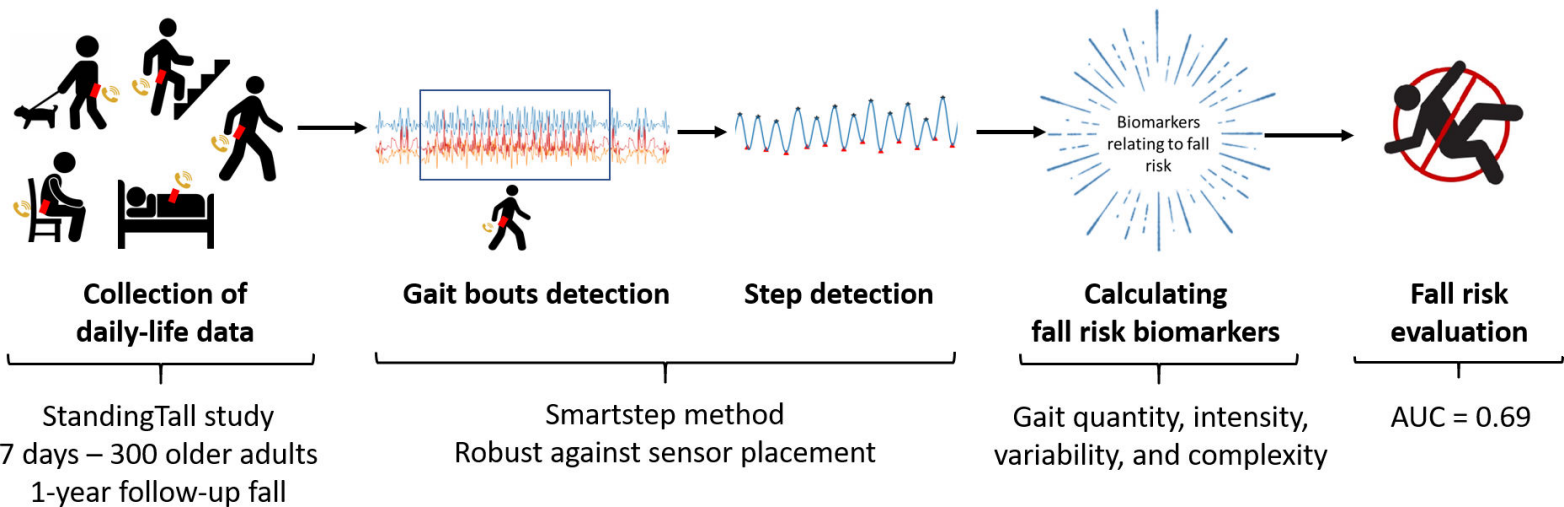
Overview of the approach used to build a fall risk prediction model based on ambulatory inertial measurement unit data. AUC: area under the curve.

An overview of the whole approach is depicted in Figure 1 [24,26].

### Data Set

This study analyzed ambulatory inertial data from the *StandingTall* study, which is described by Delbaere et al [26]. The data set includes valid inertial sensor data of 301 older people aged >70 years, who are independent in activities of daily living and without cognitive impairment, progressive neurological disease, or any other unstable or acute medical condition precluding exercise. Half of the participants were assigned to an intervention group and followed a balance-exercise program (StandingTall), whereas the other half were assigned to a health education control group. Participants wore a triaxial accelerometer (DynaPort MoveMonitor, McRoberts) for 1 week. This accelerometer had a sample rate of 100 Hz and was worn tightly on the lower back using an elastic belt. Participants were instructed to wear the accelerometer at all times, except during aquatic activities such as swimming or showering. Prospective fall incidences were obtained over a 12-month follow-up period using weekly fall diaries through a tablet computer. A fall was defined as “an unexpected event in which the participant comes to rest on the ground, floor or lower level” [27]. Table 1 summarizes the demographic characteristics of the participants.

**Table 1.** Demographic characteristic of the participants.

Characteristics	Fallers (n=116)	Nonfallers (n=185)
Age (y), mean (SD)	78 (5)	77 (5)
Female, n (%)	81 (69.8)	122 (65.9)
BMI (kg/m²), mean (SD)	28 (6)	28 (5)

### Walking Bout and Step Detection

Nonwear periods were removed using the algorithm of van Hees et al [28], and sedentary or inactive periods were removed using the accelerometer cutoff points based on the study by Migueles et al [29]. Walking bouts were then detected in 2 steps. First, Smartstep [24] was used to detect steps. Then, the detected steps were grouped into a walking bout if they were separated by less than 2 seconds. Only walking bouts longer than 200 steps, or 120 seconds of walking on average, were considered in this study. This criterion was chosen for different reasons: (1) shorter walking bouts might lead to inconclusive results [30,31], and (2) the application of some algorithms to calculate certain fall risk parameters requires a minimum number of data points [32,33].

### Fall Risk Parameters

A total of 12 *step-based* fall risk parameters were calculated. They describe four main gait domains:

1. Gait quantity: the total number of walking bouts, total number of steps, and average number of steps per walking bout
2. Gait intensity: cadence, step time, and stride time
3. Gait variability: coefficient of variance of stride time and step time
4. Gait complexity: fractal exponent and sample entropy calculated on stride and step time series

The initiation and termination phases of gait in each walking bout were discarded by removing the first 5 and last 5 steps of all walking bouts. Gait variability, intensity, and quantity parameters were calculated considering all remaining steps of the walking bouts. Gait complexity parameters were calculated on the middle 200 steps (ie, longer walking bouts were cut into a constant length of 200 steps), because the gait complexity parameters used (fractal exponent and sample entropy) are known to be dependent on the number of data points considered. Using different numbers of data points between walking bouts and participants would lead to inconsistent results.

### Statistical Analysis

#### Correlation Between Fall Risk Parameters

For each participant, we estimated the median of all fall risk parameters over all walking bouts. Spearman correlations were performed to assess the relationship between the fall risk parameters. Correlation coefficients between 0.7 and 1.0 were considered strong, between 0.4 and 0.7 were considered moderate, and between 0 and 0.4 were considered weak.

#### Statistical Model

We used a negative binomial regression model to assess the relationship between fall risk parameters and the occurrence of falls (being a faller vs nonfaller) during the follow-up period. We corrected for inclusion in the intervention group as a covariate to cancel out its potential effect. Different combinations of uncorrelated gait parameters were chosen as candidates for the model.

The interaction between gait quantity and other exploratory parameters was also considered because earlier research showed that such interactions may exist [8]. Parameters that gave the best performance were retained. Then, a backward elimination of these parameters was done in the following way. Using cross-validation, the data were split into 10 different subsets, each containing 70% of the population. A model was built on each subset. A parameter was retained if it was stable across all 10 models (ie, the mean and SD of *P* values through the models’ coefficients were less than .20). This means that the parameter maintained its stability for every chosen subset. In addition, the interaction of group allocation with the selected gait parameters were added to control for the potential effect of the intervention on the predictive ability of gait for falls.

#### Evaluation of Model

The model was evaluated using 2 methods. First, a training-testing split evaluation involved training and testing the model on the same data set. This approach was chosen for the ease of comparison with existing literature. Second, a repeated learning-testing cross-validation [34] was used for a more robust assessment. In this process, the data set was randomly divided into an 80% training set and a 20% testing set. This division was repeated 10 times and the results from each cross-validation were averaged. Concerning cross-validation, the *ShuffleSplit* method from the Python *scikit-learn* package (Python Software Foundation) was used. At each fold, the method shuffled the data set and split it into a 80% training group and a 20% testing group. The model was created or tuned in the training group and evaluated in the testing group. The AUC was calculated at each stage. The model’s performance and stability were evaluated through the mean and SD of the AUC.

### Ethical Considerations

No ethical approval was required since data for this study were obtained from a previous study (StandingTall study [26]), whose reanalysis is covered by its ethics approval.

## Results

**Figure 2.**
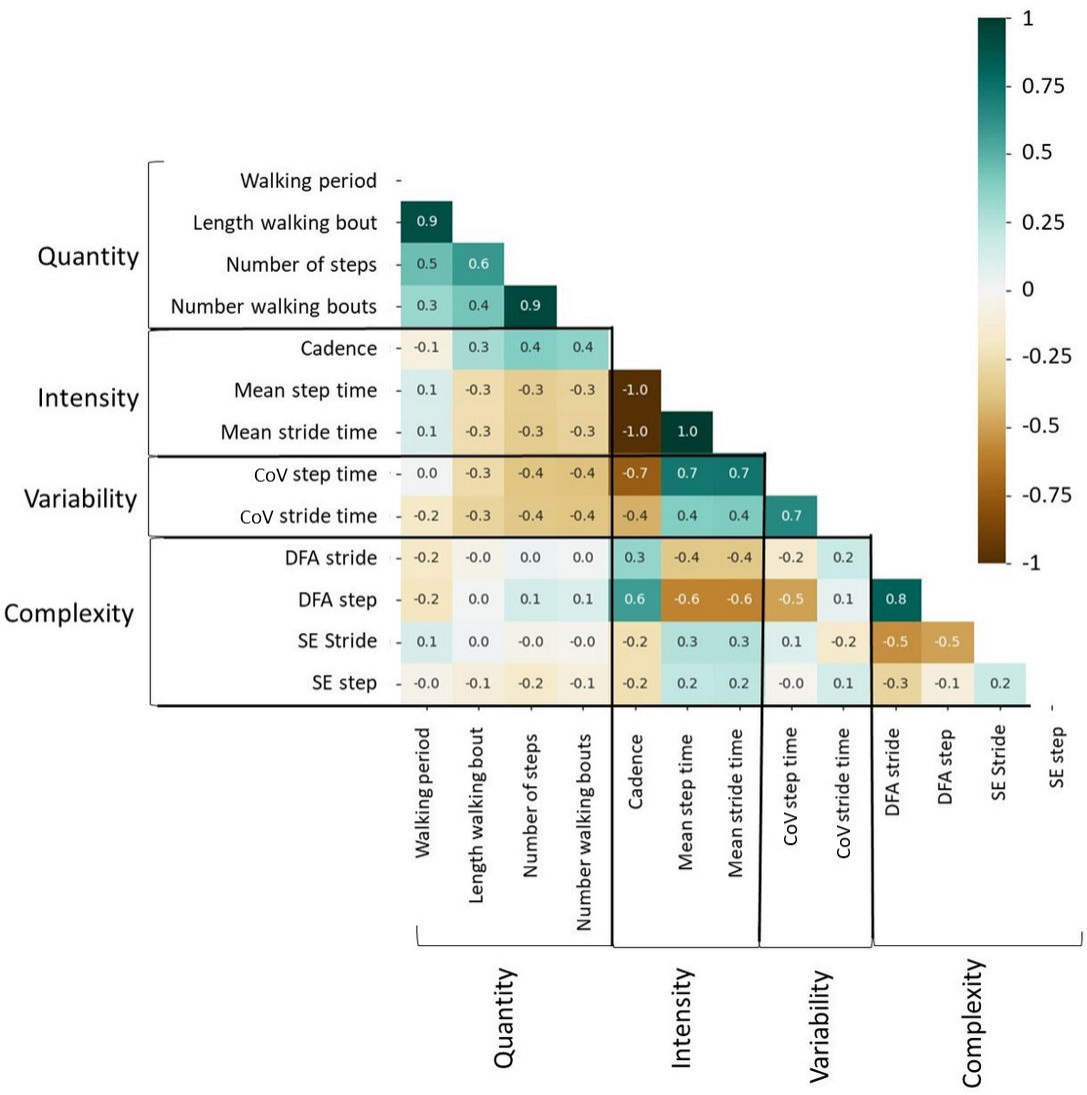
Correlation heat map between the different fall risk parameters. CoV: coefficient of variance; DFA: detrended fractal analysis; SE: sample entropy.

The selection of long walking bouts (>200 steps) caused the exclusion of 6% (18/301) of the population. The correlation heat map between fall risk parameters is shown in Figure 2. Gait quantity had moderate correlations with gait intensity and variability. Gait intensity had moderate correlations with gait complexity and strong correlations with gait variability. Finally, gait complexity and variability were not correlated.

The final fall prediction model included the total number of steps, cadence, coefficient of variance of stride time, fractal exponent on step time, sample entropy on stride time, and sample entropy on step time. The AUC of this model, which was trained and tested on the whole data set, was 0.69. The cross-validated AUC was 0.67 (SD 0.05). The model can be accessed via a public data set [35].

Table 2 displays the coefficients and *P* values of the fall risk parameters based on *z*-transformed data. All parameter coefficients were statistically significant at *P*<.05. The likelihood of being a faller increased with lower gait complexity, higher gait variability, higher gait quantity, and higher gait intensity.

**Table 2.** Multivariable negative binomial regression of selected falling risk parameters built with the whole population at a minimum of 200 steps. *B* coefficient and *P* values were based on *z*-transformed data. The area under the curve was 0.69.

Gait domain and parameter		*B *coefficient	*P* value
**Quantity**			
	Number of steps	0.20	.03
**Intensity**			
	Cadence (steps/min)	1.50	.02
**Variability**			
	CoV^a^ of stride time	0.43	<.001
**Complexit**y			
	DFA^b^ on step time	−1.79	.02
	SE^c^ on step time	−1.08	.04
	SE on stride time	−1.30	.04

a CoV: coefficient of variance.

b DFA: detrended fractal analysis.

c SE: sample entropy.

## Discussion

### Principal Findings

To our knowledge, this is the first study that used *step-based* parameters, obtained with a ubiquitous step detection method, to predict fall risk based on a large-scale, real-life gait monitoring data set.

First, the developed model demonstrated an AUC of 0.69 when tested and trained on the same data set and an AUC of 0.67 (SD 0.05) with cross-validation. These results are comparable to prediction models built solely on ambulatory data collected with a single inertial sensor (AUC=0.68-0.74) [8,10,15].

Unlike these models that rely on *signal-based* parameters—that is, requiring a fixed sensor placement, usually on the lower back—our proposed model relies on *step-based* parameters. These parameters can reliably be estimated independently of the sensor placement using Smartstep, a step detection algorithm whose robustness against sensor placement, population, and walking conditions has been demonstrated in previous validation studies [24,25]. This represents a great improvement in terms of acceptability and potential dissemination of such monitoring approaches. Furthermore, the integration of simple initial clinical assessments into these models can enhance their performance [8].

The coefficients obtained from our model (see Table 2) are in line with previous findings, suggesting that older people at high risk of falls have lower gait complexity (as indicate by negative regression coefficients for detrended fractal analysis on step time, sample entropy on step time, and sample entropy on stride time) and higher gait variability and gait quantity (as indicated by positive regression coefficients for coefficient of variance of stride time and the number of steps, respectively). These findings are consistent with previous studies on fall risk and pathological gait [8,22,36,37]. However, the positive regression coefficient for gait intensity (cadence) seems to show that higher gait intensity would be associated with a higher risk of falling, which was not reported in the literature. It is important to further investigate whether this result is a modeling artifact due to the simplicity of our model [38] (eg, potential nonlinearity or interactions with another parameter were not included) or whether this is a meaningful finding.

The correlation matrix suggested that gait variability in real life is associated with cadence or gait intensity, similar to previous findings in laboratory settings [39]. Gait complexity was also correlated with gait intensity, which aligns with the effect of gait speed on complexity in other studies [40]. The correlation between such parameters has not been studied before in ambulatory gait settings. This study provides a foundation for future studies using such variables in ambulatory settings.

### Limitations

This study has several limitations. First, the inertial data were collected from a fixed body placement on the lower back. We expect the Smartstep step detection method to be robust against sensor placement, as previously demonstrated [24,25]. However, future research should confirm this by carrying out a similar study with a different or uncontrolled sensor placement. Second, the study excluded short walking bouts (<200 steps), which in turn excluded individuals who do not walk long enough (18/301, 6% of the population). This walking period (approximately 2 min) cannot be achieved within the home. Thus, it obliges the person to go for walks outdoors, likely selecting more fit and active older people. Future studies should focus on including and analyzing short walking bouts and identifying fall risk parameters relevant to those with short walking bouts. Another limitation comes from the use of a relatively simple model (negative binomial regression) to link the occurrence of falls with the fall risk parameters. This traditional approach might have limitations in capturing more complex relationships between risk factors and occurrence of falls (such as nonlinear relations or critical thresholds) and, thus, could impede the understanding of individual effects of multiple related parameters [38]. Although the primary objective of this study was to demonstrate the feasibility of fall prediction from accelerometric data independent of the sensor placement, the logical next step would be to further investigate the modeling approach. This could include investigating data aggregation [41] or using more advanced meta-modeling approaches [38]. Furthermore, as the generalization of fall prediction models has been identified as a known challenge [13,42], it would be interesting to test the resulting fall prediction model on a new cohort.

### Implications and Future Work

The results of this study suggest that fall risk parameters can be monitored if a robust step detection algorithm is applied. This study provides the groundwork for using consumer-grade devices for fall risk monitoring. Future work can include two main topics: (1) improving the fall prediction model and (2) implementing and testing the approach on consumer-grade devices.

Several tracks could be considered to improve the fall prediction. Adding health data (eg, history of falls, results of clinical tests, and questionnaires) should further increase the model’s performance. Other propositions include adding parameters related to turning quality, GPS position, and gait spatial parameters to enhance fall prediction. More advanced modeling approaches could also be investigated.

Technical aspects and user acceptability are important considerations for implementing the approach on consumer-grade devices. The initial challenges to be tackled relate to the computation capacity, memory requirements, and battery consumption of consumer-grade devices. An option that we are currently considering is running the step detection algorithm in real time, detecting step instants, and saving them to the device for further analysis.

Finally, in this study, we focused primarily on predicting the risk of falls from a 1-week record period. An auspicious perspective offered by the proposed ubiquitous approach would be to monitor the evolution of the risk of falls or of chosen fall risk parameters through long periods and bring more insights into how they build up toward a fall event.

### Conclusion

Our study addresses the limitations of traditional fall risk assessments by proposing a set of step-based fall risk parameters that can be obtained independently of sensor placement. Our results demonstrated that the proposed parameters were comparable to models using fixed sensor placement. Specifically, higher gait variability, intensity, and quantity were associated with an increased fall risk, whereas lower gait complexity was also identified as a significant factor. These findings highlight the potential of our method for comprehensive and accurate fall risk assessments, independent of sensor placement, thus offering promising implications for ambulatory gait monitoring and fall prevention strategies.

## Abbreviations

AUC: area under the curve
